# Effects of Ultrasound Technique on the Composition of Different Essential Oils

**DOI:** 10.1155/2019/6782495

**Published:** 2019-04-28

**Authors:** Radosław Kowalski, Mariusz Gagoś, Grażyna Kowalska, Urszula Pankiewicz, Monika Sujka, Artur Mazurek, Agnieszka Nawrocka

**Affiliations:** ^1^Department of Analysis and Evaluation of Food Quality, University of Life Sciences in Lublin, 8 Skromna Street, 20-704 Lublin, Poland; ^2^Department of Cell Biology, Institute of Biology and Biochemistry, Maria Curie–Skłodowska University, 20-033 Lublin, Poland; ^3^Department of Tourism and Recreation, University of Life Sciences in Lublin, 15 Akademicka Street, 20-950 Lublin, Poland; ^4^Bohdan Dobrzański Institute of Agrophysics Polish Academy of Sciences, Doświadczalna 4, 20-290 Lublin, Poland

## Abstract

The objective of the experiment was to investigate the stability of the composition of selected essential oils in the model systems containing methanol and hexane solutions which were treated with ultrasound. Solutions of the oils, with a concentration of 90 mg/ml, were subjected to the effect of ultrasounds with a frequency of 20 kHz and an output power of 200 W for periods of 2 min and 10 min at 50% and 80% power. The experiment has shown no significant effect on the composition of the essential oils resulting from the applied parameters of the process in the tested model systems. The study indicates that the sonication parameters adopted in the experiment can be applied in the case of analogous systems containing essential oils in their composition.

## 1. Introduction

Essential oils are aromatic substances, mixtures of various organic compounds. Approximately 300 essential oils are manufactured on an industrial scale, but it is estimated that there are approximately 18 thousand oil-bearing plant species in existence on Earth. Each essential oil has its specific effect (bactericidal, moistening, and medicinal) and individual flavour (citrus, spicy, floral, and herbal). The range of application of essential oils has expanded notably since they ceased to be confined to specialist laboratories and are now available to the general public via pharmacies and herb shops [1–3].

Today, essential oils are becoming more and more popular as alternative substances used for improving the quality of food products, i.e., stability or sensory parameters (taste and smell). These ingredients can be directly added during the process of food production either as pure mixtures or in the form of stabilized lipophilic or hydrophilic solutions. In technological applications, methods allowing to obtain the highest quality product are used. The applied technological factors, such as physical parameters of the process, can have a negative effect on the molecular structure of chemical components in food, as they may accelerate their degradation or initiate unfavourable chemical changes. They can also affect the releasing of volatile compounds from the food matrix, which can take place, e.g., in the case of essential oils. One of the known physical factors used in food processing is ultrasound, which is applied mainly in order to intensify the secretion of active ingredients (extraction and support of the distillation process) but can also facilitate the mixing of ingredients (emulsification and dissolution) or influence the product structure [4]. Modified methods may be more efficient in the case of “difficult” raw materials; for example, distillation or extraction supported by ultrasound can be useful for hard plant materials (seeds, roots, and wood).

For separating volatile biologically active substances (essential oil), the most well-known classical processes are used, i.e., distillation, rectification, pressing, extraction with various solvents, maceration, and extraction with liquid gases in the supercritical state [5–7]. Currently, research is being conducted on the application of physical factors, e.g., ultrasounds or microwaves, for the purpose of augmentation of the classic methods used so far in production processes [8–11]. Condurso et al. [11] show that Sicilian red garlic dried by the microwave drying technique was richer in the volatile fraction compared to that dried with the use of the traditional technique. Moreover, this innovative product was preferred by the consumers. The economic aspect of such modifications is also important because it determines whether their applications will be financially viable. Literature data indicate that the technique of distillation, augmented with ultrasounds, is characterised by a higher efficiency factor compared to the classic distillation technique [9, 10, 12]. According to Assami and coworkers [12], sonication of caraway seeds (*Carum carvi*) prior to distillation improved the efficiency in terms of the amount of essential oil acquired which allowed to shorten the distillation stage. Shortening the process of extracting essential oils from orange peel by applying an ultrasonic horn did not result in any significant loss in their quality when compared to the conventional method in situ [13]. Moreover, in earlier studies, it was found that the composition of essential oil distilled using the method of ultrasonic augmentation differed from the composition of essential oil distilled with the classic method. The authors of those studies attribute the changes in oil efficiency as well as the qualitative changes to the fact of higher effectiveness of sonication in the extraction process of those substances from the secretory structures of plants. They suppose that certain unstable chemical compounds may undergo, e.g., isomerisation under the effect of ultrasounds [14, 15].

In the case of extracting an essential oil on an industrial scale, the distillation method is now the principal method. Of course, classical methods are not free from factors that may result in adverse changes to the composition of the obtained essential oil. In addition, the introduction of new physical factors, i.e., ultrasounds, may provoke a number of further significant and undesirable changes in the composition of the essential oils, such as isomerisation, oxidation, and polymerisation. Moreover, it is interesting whether the use of ultrasound in technological processes can interact at the molecular level with the structures of volatile compounds present in various systems, i.e., solvent–essential oil systems or food matrix–essential oil systems.

Therefore, the aim of the experiment was to study the stability of the composition of selected essential oils in the model systems containing methanol and hexane solutions which were treated with ultrasound.

## 2. Experimental

### 2.1. Experimental Material

The experimental material consisted of commercial natural essential oils from the series “Dr Beta” (FSZ Pollena-Aroma, Warsaw, Poland): oils of lemon balm (*Melissa officinalis* L.), basil (*Ocimum basilicum* L.), chamomile (*Matricaria chamomilla* L.), hyssop (*Hyssopus officinalis* L.), rosemary (*Rosmarinus officinalis* L.), peppermint (*Mentha × piperita* L.), and marjoram (*Origanum majorana* L.).

### 2.2. Preparation of Solutions of Essential Oils

900 mg portions of the particular essential oils were weighed and placed in calibrated flasks with volume of 10 ml. Next, the flasks were filled up to the mark with methanol (methanol solutions) or hexane (hexane solutions) with HPLC purity, thus obtaining the initial concentrations of the oils of 90 mg/ml. The solutions of essential oils were prepared in triplicate.

### 2.3. Preparation of Solutions of Internal Standards

250 mg of the following standard substances were weighed and placed in ground glass measuring flasks with volume of 25 ml: dodecane (±99%, Sigma-Aldrich Chemic GmbH, Germany) and nonadecane (±99%, Sigma-Aldrich Chemic GmbH, Germany), topping up to the mark with methanol or hexane, respectively, with HPLC purity.

### 2.4. Preparation of Solutions of Main Components of the Oils

The standard solutions were prepared in accordance with a procedure described earlier [10]. Samples (15.0 mg) of individual substances (terpinene <gamma->, linalool, 1,8-cineole, menthone, limonene, Sigma-Aldrich) were weighed to measuring flasks with ground glass of 10 ml capacity and made up to volume with methanol (HPLC grade) to give the concentration of the respective components at a level of 1.5 mg/ml.

### 2.5. Sonication of Essential Oil Solutions

Two-milliliter portions of methanolic and hexane solutions of essential oils and standard solutions were transferred to a vial of ultrasonic disintegrator. Vials were placed in an ice bath (4°C) preventing sample heating. The rod of the ultrasonic disintergrator-type HD 2200 (Bandelin Electronic, Berlin), with an operation frequency of 20 kHz and output power of 200 W, was inserted into the solutions through the aperture of the vial. The solutions of the essential oils were subjected to the effect of ultrasounds with various power levels and for different times (Table 1).

Next, the portions of 1100 *μ*l of solution were taken from each of the samples subjected to sonication (A, B, C, and D) and from the control sample (K) and transferred to analytical vials. Then, 10 *μ*l of nonadecane (10 mg/ml) and dodecane (10 mg/ml) solutions were added as internal standards. The samples were used for chromatographic analysis.

### 2.6. Chromatographic Analysis

The chromatographic analysis was performed according to procedures described previously [10]. The analysis was performed in triplicate.

#### 2.6.1. GC-MS

The essential oils were analysed using a Varian 4000 GC-MS/MS system (Varian, USA). The compounds were separated on 30 m × 0.25 mm × 0.25 *μ*m VF-5 ms column (Varian, USA). The column temperature was increased from 50°C to 250°C at a rate of 4°C/min; injector temperature 250°C; split ratio 1 : 50; and injection volume 5 *μ*L. MS parameters were as follows: EI mode, with ionization voltage 70 eV, ion source temperature 271.2°C; scan range 40–870 Da, scan time 0.80 s, manifold temp. 45°C, and transfer line temp. 289.5°C.

#### 2.6.2. GC-FID

The essential oils were analysed using a GC Varian 3800 system GC-FID (Varian, USA). The compounds were separated on 30 m × 0.25 mm × 0.25 *μ*m DB-5 column (J&W Scientific, USA). The GC program was the same as those used for GC-MS analysis.

#### 2.6.3. Qualitative and Quantitative Analysis

The qualitative (MS spectra and retention indices analysis) and quantitative analysis (internal standard addition method—alkanes C12 and C19) were performed according to previously described procedures [9]. Precision was estimated by evaluating intraday (repeatability) and interday precision in accordance with a procedure described earlier [16]. Determination of intraday precision was carried out by triplicate injection of terpinene <gamma->, linalool, 1,8-cineole, menthone, and limonene solutions of three different concentrations (0.5, 5, and 20 mg/ml). The same three samples were injected over three consecutive days in order to assess interday precision. Inter- and intraday precisions were expressed as the relative standard deviation (RSD) and were within an acceptable range of 2.5% to 4.7%.

### 2.7. Statistical Analysis

Data were analysed by analysis of variance (Duncan's test) at 5% significance level using the SAS statistical system (SAS Version 9.1, SAS Inst., Cary, NC, USA).

## 3. Results and Discussion

Chromatographic analysis of methanol and hexane solutions of basil oil revealed the presence of 13 main compounds (Table 2). The highest content in the solution of the analysed oil was that of methyl chavicol (from 62.18 mg/ml (control sample in methanol) to 64.40 mg/ml (oil in methanol subjected to sonication for 2 minutes at 80% power)). In terms of the amount, linalool was the second dominant component (from 10.61 mg/ml (oil in hexane subjected to sonication for 2 minutes at 80% power) to 11.78 mg/ml (oil in hexane subjected to sonication for 2 minutes at 50% power)). Statistical analysis of the results obtained for the dominant components revealed that the sonication applied had no significant effect on the concentration of volatile compounds in the analysed solutions. Basil cultivars grown in Europe are characterised by the occurrence of linalool as the main component of essential oil (from 46.9% to 71.8%), while those grown in the tropical climate contain methyl chavicol as the main component (up to 94.3%). Oil obtained from basil grown in Poland contained up to 69.7% of linalool and up to 12.2% of geraniol [18]. Dolatabad et al. [19] reported that the main components of basil oil acquired from cultivars grown in Iran were methyl chavicol (up to 43.0%) and linalool (up to 33.4%).

GC analysis of methanol and hexane solutions of marjoram oil revealed the presence of 28 main compounds (Table 3). The highest concentration in solutions of marjoram oil was the characteristic of terpinene-4-ol: from 14.50 mg/ml (oil in methanol subjected to sonication for 2 minutes at 80% power) to 15.31 mg/ml (oil in methanol not subjected to sonication). Other dominant components in the analysed solutions of marjoram oil included terpinene <gamma-> (up to 10.62 mg/ml) and sabinene hydrate <*trans*-> (up to 9.62 mg/ml). Statistical analysis of the results obtained showed that the sonication applied in the experimental systems did not have any significant effect on the levels of the main components identified. The oil composition of marjoram grooving in India showed terpinen-4-ol (31.15%), *cis*-sabinene hydrate (15.76%), sabinene (6.91%), cymene <*para*-> (6.83%), *trans*-sabinene hydrate (3.86%), and terpineol <*alpha*-> (3.71%) as the main constituents [20].

22 main volatile compounds were identified as a result of GC analysis of methanol and hexane solutions of peppermint oil (Table 4). The highest concentration in the analysed solution was noted for menthol (from 35.90 mg/ml (peppermint oil in hexane subjected to sonication for 2 minutes at 50% power) to 37.34 mg/ml (peppermint oil in hexane subjected to sonication for 2 minutes at 80% power)). The second dominant component in terms of its concentration was menthone (from 16.88 mg/ml (peppermint oil in methanol subjected to sonication for 2 minutes at 50% power) to 18.37 mg/ml (peppermint oil in hexane subjected to sonication for 2 minutes at 50% power)). Statistical analysis of the results obtained showed that the sonication applied in the experimental systems did not have any significant effect on the levels of the main components identified. Until now, approximately 150 components of peppermint essential oil have been identified, the main ones being menthol (20–80%) and menthone (15–45%) [21]. Mckay and Blumberg [22] reported that the dominant components of *M. piperita* oil are menthol (33–60%), menthone (15–32%), 1,8-cineole (5–13%), menthyl acetate (2–11%), isomenthone (2–8%), menthofuran (1–10%), limonene (1–7%), myrcene <*beta*-> (0.1–1.7%), caryophyllene <*beta*-> (2–4%), pulegone (0.5–1.6%), and carvone (1%).

Chromatographic analysis of methanol and hexane solutions of chamomile oil revealed the presence of 25 main compounds (Table 5). Dominant concentration in the analysed solution was the characteristic of bisabolol oxide A <*alpha*-> (from 30.97 mg/ml (chamomile oil in methanol subjected to sonication for 2 minutes at 50% power) to 33.16 mg/ml (chamomile oil in methanol subjected to sonication for 10 minutes at 80% power)). The main components of solutions of chamomile oil included also farnesene <(E)-*beta*-> (up to 17.24 mg/ml) and bisabolone oxide A <*alpha*-> (up to 11.28 mg/ml). Statistical analysis of the quantitative results did not reveal any significant differences in the concentration of the main components of chamomile oil for the analysed experimental systems with the use of sonication. According to the literature, the dominant components of chamomile oils are bisabolol oxide A <*alpha*-> (2%–60%), bisabolol <*alpha*-> (1–60%), bisabolol oxide B <*alpha*-> (3–50%), E-*β*-farnesene (5–40%), chamazulene (2–25%), and bisabolol oxide A <*alpha*-> (0.4–12%), which finds support in the results of the study presented here [21, 23–25].

In methanol and hexane solutions of hyssop oil, the presence of 31 main volatile components was noted (Table 6). The dominant component in the analysed solutions was pinocamphone <*cis*-> with content varying from 27.17 mg/ml (hyssop oil in hexane subjected to sonication for 2 minutes at 80% power) to 29.38 mg/ml (methanol solution of hyssop oil subjected to sonication for 2 minutes at 80% power). The second main component of hyssop oil solutions was pinene <*beta*-> (up to 13.96 mg/ml). Statistical analysis did not reveal any significant effect of sonication parameters applied in the experiment on changes in the concentration of the main components of hyssop oil. The total content of the main identified components fell within the range from 85.20 mg/ml to 86.48 mg/ml. According to Džamić et al. [26], the main components of hyssop oil are 1,8-cineole (36.43%), pinene <*beta*-> (19.55%), and pinocamphone <*cis*-> (15.32%). Essential oil from plants cultivated in Italy contains mainly pinocamphone <*cis*-> with a content of 43.3% and limonene with a content of 12.2% [27], whereas, in hyssop oil from Bulgaria, the main components were pinocamphone <*cis*-> (48.98%) and pinene <*beta*-> (13.54%) [28].

GC analysis of methanol and hexane solutions of lemon balm oil confirmed the presence of 22 main compounds (Table 7). The highest concentration in the analysed solutions of lemon balm oil was the characteristic of citronellal, with levels from 16.38 mg/ml (methanol solution of lemon balm oil not subjected to sonication—control) to 17.44 mg/ml (lemon balm oil in hexane subjected to sonication for 10 minutes at 50% power). Other dominant components of the oil included the following volatile compounds: nerol (up to 11.92 mg/ml), caryophyllene <E-> (up to 9.07 mg/ml), limonene (up tp 7.60 mg/ml), and neral (up to 8.16 mg/ml). Statistical analysis of the quantitative results obtained revealed that the sonication applied had no effect on the content of the identified main components. Generally, the total concentration of the main components in methanol and hexane solutions of lemon balm oil varied from 80.56 mg/ml to 82.87 mg/ml. According to Góra and Lis [21], the qualitative composition of lemon balm oil is subjected to notable variation. Large differences were observed in the levels of the main components, which is supported by the results of this study. The authors cited state that the dominant components of lemon balm oil are geranial (33.6%), neral (22.18%), and citronellal (11.31%). In lemon balm oils obtained from plants grown in Poland, the following main components were assayed: geranial (up to 32.92%), caryophyllene <*beta*-> (up to 31.73%), neral (up to 17.37%), citronellal (up to 15.18%), and caryophyllene oxide <*beta*-> (up to 12.2%) [29]. In essential oils isolated from lemon balm grown in Morocco, 33 components were identified, the dominant ones being citronellal (14.40%), caryophyllene oxide (11.00%), geraniol acetate (10.20%), E-caryophyllene (8.10%), isogeraniol (6.40%), and nerol acetate (5.10%) [30].

Chromatographic analysis of methanol and hexane solutions of rosemary oil revealed the presence of 19 main components (Table 8). Dominant concentration in the analysed solutions was that of cineole <1,8-> with levels from 19.06 mg/ml (hexane solution subjected to sonication for 10 minutes at 50% power) to 20.75 mg/ml (methanol solution subjected to sonication for 10 minutes at 50% power), with camphor being the second dominant component in terms of quantity, its concentration varying from 12.70 mg/ml (rosemary oil in hexane subjected to sonication for 2 minutes at 80% power) to 13.93 mg/ml (hexane solution of rosemary oil subjected to sonication for 10 minutes at 80% power). Statistical analysis of the quantitative results did not reveal any significant changes in the composition of volatile compounds for solutions subjected to sonication. In general, the total content of the particular identified components varied from 87.30 mg/ml to 89.94 mg/ml. Zaouali et al. [31] reported that the main components of rosemary oil were 1,8-cineole (35.80%), camphor (14.50%), pinene <*alpha*-> (10.10%), and borneol (9.20%).

Comparing the composition of alcohol and hexane solutions of essential oils subjected to sonication with that of the control solutions, one can observe only statistically insignificant quantitative differences that result from the uncertainty of the analytical procedure. The experiment demonstrated that parameters of sonication of essential oil solutions applied in the experiment had no significant effect on the composition of these essential oils, which is important in the aspect of qualitative evaluation and has a bearing on the requirements regulated by relevant standards (pharmacopeia). The study shows that the sonication parameters adopted in the experiment can be applied in the case of analogous systems containing essential oils in their composition. The use of physical methods augmenting the processes of isolation is often determined by the stability of those systems at predetermined parameters. The stability of a system, in turn, is related with an absence of quantitative changes among the desirable components that determine the biological activity of the final product. The choice of a suitable extraction method is extremely important since the oil quality is related to its chemical composition. To maintain the quality of the final product, the applied method should not influence negatively its composition; e.g., the interesting compounds should not be decomposed [2, 32].

There are few research reports that address the problem of using the ultrasound technique for the extraction of aromatic substances from the plant raw material. Experiments conducted so far demonstrated that ultrasounds can effectively cause an increase in the efficiency of the process of distillation of essential oils [9, 10, 12, 13, 33]. The cited authors explain that ultrasounds enhance the extraction of aromatic substances from secretory structures of plants, which also takes place in the case of the process of extraction with the use of various solvents [34]. In addition, Kowalski and Wawrzykowski [9] and Da Porto and Decorti [35] demonstrated that composition of oils distilled from the plant material treated with ultrasounds differed from oils obtained with the classical methods. The differences in the quantitative composition of oils obtained with methods augmented with ultrasounds may result from the fact of more effective liberation of certain chemical components from the secretory cells [9, 35], as well as from structural transformations of unstable chemical compounds in the secretory cells (decomposition of dimers and polymers, oxidation, and isomerisation) [9, 14, 15]. The results of the study presented here provide evidence that the sonication applied in the experiment did not have any significant effect on quantitative changes among the main volatile components, which justifies the conclusion that any quantitative changes in oil distilled with sonication are related rather with increased efficiency of extraction of the components from hard-accessible anatomical structures.

Much more extensive was the research in the area of extraction augmentation. Da Porto and Decorti [35] reported that the application of ultrasounds in ethanol-water extraction (70%) of mint had a significant effect on the content of individual oil compounds distilled from that extract, compared to the composition of oil obtained with the method of classic direct distillation. The above authors concluded that oil acquired with the pharmacopeial method was poorer quantitatively in oxidised monoterpenes (ca. 4097 *μ*g/g of fresh matter of plant) compared to the content of oxygen-derived monoterpenes in oil distilled from the fraction of extracts obtained with ultrasonic augmentation (from ca. 20174 *μ*g/g to ca. 33731 *μ*g/g of the fresh plant matter). Probably, one of the causes of the above quantitative changes was sonication that may accelerate the processes of oxidation, catalysing the formation of reactive forms of oxygen and that may initiate the processes of oxidation. In the results of the presented study being the object of discussion it was not observed that ultrasounds had an effect causing a significant increase in the content of oxygen-derived oil substances.

In general, the time of extraction was shortened by three times (mean average) when ultrasound was used for pretreatment of the samples. Nevertheless, the increase of the rate of bioactive compounds and normally present some changes in the chemical composition provide greater features to apply these extracts as natural antioxidants. The increased quality of the essential oils can be attributed to the low level of degradation of thermal compounds [10, 12, 13]. The minimal degradation of the chemical compounds refers mainly to the low submission time of the plant material to heating. Moreover, essential oils are released faster because ultrasound action breaks the cell membranes of the glands. This phenomenon enables an effective extraction in a considerable shorter time with a better quality in comparison to conventional methods [13, 36]. The results obtained in this study are promising, but it should be considered that this technique is not always appropriate, as indicated in the work cited by Da Porto and Decorti [35], among others. The changes and differences observed may be caused by a competition between oxidations or other reactions promoted by heating and water acid medium in distillation, and those caused by sonication, acting by different physicochemical mechanisms.

The phenomenon of formation, expansion, and disappearance of bubbles or other small enclosed spaces in a solution, containing vapour, gas, or a vapour-gas mixture, taking place under suitable temperature-pressure conditions under the effect of ultrasounds (cavitation) is well known. In a water environment, with the participation of ultrasounds, one can observe the formation of reactive forms of H˙, OH˙, and H_2_O_2_ that may interact with numerous chemical substances, leading to their transformation (e.g., decomposition) [37]. Especially susceptible to such effects are organic volatile compounds that may undergo various reactions in a cavitation “bubble.” The experiment conducted in the study presented here did not demonstrate any significant quantitative changes taking place in methanol or hexane solutions of standards of oil compounds (Table 9). Chromatographic analysis of the standard solutions did not prove any effect of ultrasounds on transformation or decomposition of the selected substances—in the systems subjected to sonication, no presence was found of other substances that would be registered as chromatographic peaks at the attenuation used, in comparison to the control systems. This allows the conclusion that the application of sonication with technical parameters corresponding to those applied in the experiment does not cause unfavourable quantitative changes in methanol and hexane solutions in relation to the individual components of the volatile fraction.

## 4. Conclusions

The results of the essential oils analysed revealed that the applied sonication parameters did not affect significantly the composition of essential oils in the tested model systems containing their methanol or hexane solutions. The sonication parameters adopted in the experiments should be used with other aromatic species, in order to assess if the quality of the essential oils obtained are allowable.

## Figures and Tables

**Table 1:** Statement of sonication time and power levels.

Sonication parameters
A: 2 minutes at 50% power
B: 2 minutes at 80% power
C: 10 minutes at 50% power
D: 10 minutes at 80% power
K: control sample not subjected to sonication

**Table 2:** Changes in the chemical composition in solutions of basil oil.

Compound	IR	Chemical composition of essential oil (mg/ml)									
		Alcohol solutions					Hexane solutions				
		A^*∗*^	B	C	D	K	A	B	C	D	K
Pinene <*alpha*->	939	0.89	0.81	0.70	0.84	0.89	1.06	1.09	1.00	1.12	1.05
Pinene <*beta*->	981	0.57	0.39	0.45	0.42	0.44	0.68	0.70	0.63	0.72	0.66
Cineole <1,8->	1033	5.41^a^	5.40^a^	5.55^a^	5.57^a^	5.70^a^	5.97^a^	6.11^a^	5.72^a^	6.10^a^	5.94^a^
Linalool	1099	11.78^a^	11.29^a^	11.63^a^	11.67^a^	11.52^a^	11.07^a^	10.61^a^	10.74^a^	11.56^a^	10.89^a^
Menthol	1172	0.24	0.33	0.43	0.52	0,47	0.54	0.57	0.52	0.53	0.63
Methyl chavicol	1200	63.31^a^	64.40^a^	61.96^a^	62.21^a^	62.18^a^	62.26^a^	64.06^a^	63.31^a^	63.53^a^	63.79^a^
Geraniol	1254	0.29	0.29	0.38	0.31	0.32	0.31	0.32	0.38	0.36	0.37
Anethole <Z->	1255	0.27	0.25	0.28	0.24	0.25	0.35	0.36	0.31	0.35	0.36
Eugenol	1360	0.22	0.12	0.22	0.17	0.20	0.35	0.37	0.33	0.38	0.31
Methyl cinnamate <E->	1380	1.34	1.34	1.04	1.27	1.38	1.18	1.24	1.10	1.26	1.15
Bergamotene <*alpha*-*trans*->	1436	0.77	0.51	0.65	0.55	0.60	0.87	0.88	0.79	0.91	0.85
Cinnamaldehyde <E-*para*-methoxy->	1566	1.41	1.34	1.31	1.33	1.36	0.99	1.05	0.96	0.98	0.94
Cadinol <epi-*alpha*->	1642	0.22	0.12	0.18	0.14	0.18	0.22	0.23	0.20	0.24	0.22

^*∗*^Designation according to Table 1. IR: retention indices (from temperature programming, using the definition of Van den Dool and Kratz [17]). Values designated with the same superscript letters for the dominant compound do not significantly differ at 5% error (Duncan's test).

**Table 3:** Changes in the chemical composition in solutions of marjoram oil.

Compound	IR	Chemical composition of essential oil (mg/ml)									
		Alcohol solutions					Hexane solutions				
		A^*∗*^	B	C	D	K	A	B	C	D	K
Thujene <*alpha-*>	931	0.96	1.00	1.10	1.04	1.05	1.02	1.01	1.03	1.04	1.00
Pinene <*alpha*->	939	1.15	1.19	1.31	1.24	1.25	1.23	1.23	1.24	1.27	1.22
Sabinene	975	7.02	7.23	7.93	7.52	7.55	7.18	7.14	7.17	7.26	7.04
Pinene <*beta*->	982	0.59	0.61	0.68	0.64	0.63	0.63	0.64	0.64	0.65	0.63
Myrcene	994	2.17	2.3	2.46	2.34	2.34	2.24	2.23	2.26	2.29	2.23
Phellandrene <*alpha*->	1008	0.49	0.52	0.57	0.54	0.54	0.51	0.50	0.50	0.52	0.51
Terpinene <*alpha*->	1020	7.37^a^	7.18^a^	6.50^a^	6.91^a^	7.02^a^	7.49^a^	7.40^a^	7.42^a^	7.16^a^	7.30^a^
Cymene <*para*->	1026	3.01	3.2	3.40	3.25	3.26	3.14	3.06	3.06	3.09	3.00
Limonene	1030	2.33	2.46	2.65	2.45	2.53	2.33	2.35	2.34	2.35	2.38
Phellandrene <*beta*->	1031	2.29	2.33	2.54	2.46	2.42	2.27	2.17	2.20	2.25	2.14
Terpinene <gamma->	1060	10.60^a^	9.64^a^	10.18^a^	10.41^a^	10.45^a^	10.62^a^	10.36^a^	10.37^a^	10.19^a^	10.15^a^
Sabinene hydrate <*cis*->	1071	3.53	3.75	3.57	3.60	3.28	3.43	3.34	3.37	3.39	3.31
Terpinolene	1091	3.22	3.39	3.64	3.42	3.08	3.20	3.10	3.12	3.16	3.11
Linalool	1099	2.7	2.49	2.35	2.41	2.27	2.84	2.75	2.75	2.75	2.81
Sabinene hydrate <*trans*->	1102	9.62^a^	8.68^a^	8.31^a^	8.76^a^	8.80^a^	9.62^a^	9.36^a^	9.39^a^	9.50^a^	9.45^a^
Menth-2-en-1-ol <*cis*-*para*->	1122	1.65	1.79	1.32	1.34	1.70	1.56	1.53	1.55	1.57	1.57
Menth-2-en-1-ol <*trans*-*para*->	1140	1.22	1.3	1.28	1.23	1.25	1.17	1.17	1.18	1.20	1.19
Terpinen-4-ol	1178	14.49^a^	14.50^a^	14.70^a^	14.20^a^	15.31^a^	15.09^a^	14.93^a^	15.03^a^	15.24^a^	15.16^a^
Terpineol <*alpha*->	1190	4.23	4.09	4.51	4.25	4.03	3.59	3.90	3.91	3.96	3.92
Piperitol <*cis*->	1199	0.70	0.75	0.73	0.64	0.67	0.63	0.64	0.64	0.64	0.64
Citronellol	1227	0.58	0.63	0.61	0.57	0.54	0.55	0.54	0.53	0.54	0.54
Linalyl acetate	1257	2.56	2.09	2.74	2.55	2.65	2.40	2.39	2.4	2.42	2.41
Myrtanol <*trans*->	1261	0.59	0.63	0.61	0.58	0.42	0.56	0.56	0.56	0.57	0.57
Terpinen-4-ol acetate	1302	0.31	0.24	0.34	0.37	0.32	0.30	0.28	0.29	0.37	0.29
Caryophyllene <E->	1420	3.09	3.39	2.71	3.14	3.16	2.85	2.80	2.74	2.64	2.80
Bicyclogermacrene	1503	1.59	1.68	1.67	1.59	1.6	1.46	1.40	1.41	1.47	1.43
Spathulenol	1580	0.22	0.24	0.22	0.22	0.02	0.21	0.21	0.21	0.21	0.21
Caryophyllene oxide	1585	0.22	0.24	0.23	0.22	0.20	0.22	0.22	0.22	0.22	0.22

^*∗*^Designation according to Table 1. IR: retention indices (from temperature programming, using the definition of Van den Dool and Kratz [17]). Values designated with the same superscript letters for the dominant compound do not significantly differ at 5% error (Duncan's test).

**Table 4:** Changes in the chemical composition in solutions of peppermint oil.

Compound	IR	Chemical composition of essential oil (mg/ml)									
		Alcohol solutions					Hexane solutions				
		A^*∗*^	B	C	D	K	A	B	C	D	K
Pinene <*alpha*->	939	0.91	0.94	0.87	0.97	0.88	0.98	0.94	0.95	1.07	0.95
Sabinene	975	0.51	0.54	0.50	0.55	0.50	0.52	0.50	0.51	0.56	0.51
Pinene <*beta*->	981	1.17	1.23	1.15	1.28	1.15	1.27	1.23	1.24	1.40	1.24
Cymene <*para*->	1026	0.32	0.34	0.32	0.35	0.32	0.67	0.64	0.64	0.72	0.65
Limonene	1030	2.60	2.70	2.61	2.84	2.56	2.83	2.76	2.76	3.09	2.75
Cineole <1,8->	1033	4.79	4.96	4.80	5.13	4.74	5.05	4.85	4.83	5.38	4.86
Terpinene <gamma->	1060	0.20	0.21	0.20	0.22	0.20	0.22	0.22	0.22	0.25	0.21
Isopulegol	1151	0.26	0.27	0.28	0.28	0.28	0.17	0.24	0.24	0.21	0.25
Menthone	1155	16.88^a^	16.98^a^	17.12^a^	17.26^a^	17.20^a^	18.37^a^	17.72^a^	17.73^a^	17.45^a^	17.85^a^
Menthofuran	1165	3.08	3.07	3.04	3.14	3.07	2.06	2.05	2.03	2.25	2.05
Menthone <*iso*->	1164	2.89	2.95	2.90	2.94	2.89	3.24	3.10	3.12	3.55	3.19
Menthol <neo->	1167	3.05	3.07	3.13	3.07	3.17	3.98	3.870	3.87	3.48	3.93
Menthol	1174	36.93^a^	36.51^a^	36.83^a^	36.38^a^	36.39^a^	35.90^a^	37.34^a^	37.33^a^	34.23^a^	37.05^a^
Menthol <*iso*->	1184	0.22	0.22	0.22	0.22	0.22	0.14	0.280	0.27	0.31	0.14
Terpineol <*alpha*->	1190	0.28	0.28	0.29	0.28	0.30	0.22	0.220	0.22	0.25	0.23
Pulegone	1237	1.76	1.76	1.75	1.73	1.77	1.55	1.520	1.53	1.70	1.54
Piperitone	1253	0.32	0.31	0.33	0.28	0.30	0.22	0.240	0.24	0.26	0.25
Menthyl acetate <neo->	1275	0.25	0.24	0.25	0.24	0.25	0.23	0.240	0.24	0.26	0.27
Menthyl acetate	1298	6.11^a^	6.06^a^	6.11^a^	5.99^a^	6.22^a^	5.22^a^	5.06^a^	4.98^a^	5.59^a^	5.22^a^
Caryophyllene <E->	1420	4.88	4.79	4.77	4.56	4.98	3.96	3.84	3.81	4.31	3.98
Germacrene D	1487	0.48	0.46	0.47	0.42	0.50	0.10	0.09	0.09	0.11	0.10
Caryophyllene oxide	1585	0.32	0.32	0.30	0.27	0.34	1.44	1.35	1.36	1.58	1.37

^*∗*^Designation according to Table 1. IR: retention indices (from temperature programming, using the definition of Van den Dool and Kratz [17]). Values designated with the same superscript letters for the dominant compound do not significantly differ at 5% error (Duncan's test).

**Table 5:** Changes in the chemical composition in solutions of chamomile oil.

Compound	IR	Chemical composition of essential oil (mg/ml)									
		Alcohol solutions					Hexane solutions				
		A^*∗*^	B	C	D	K	A	B	C	D	K
Ocimene <(E)-*beta*->	1049	0.56	0.56	0.49	0.61	0.63	0.65	0.68	0.69	0.71	0.64
Terpinene <gamma->	1060	1.09	1.05	0.94	1.20	1.21	1.17	1.22	1.30	1.27	1.17
Copaene <*alpha*->	1377	0.26	0.29	0.22	0.28	0.38	0.90	0.94	0.53	0.82	0.72
Elemene <*beta*->	1393	0.43	0.50	0.40	0.51	0.51	0.43	0.42	0.48	0.45	0.47
Caryophyllene <E->	1420	0.27	0.30	0.22	0.28	0.29	0.27	0.28	0.31	0.35	0.26
Aromadendrene	1442	0.25	0.28	0.21	0.28	0.28	0.25	0.26	0.27	0.26	0.24
Farnesene <(E)-*beta*->	1457	16.71^a^	17.02^a^	16.22^a^	16.01^a^	16.21^a^	17.24^a^	16.21^a^	16.43^a^	17.01^a^	16.91^a^
Aromadendrene <allo->	1461	0.30	0.40	0.29	0.37	0.38	0.31	0.32	0.29	0.35	0.31
Muurolene <gamma->	1480	0.33	0.32	0.18	0.41	0.44	0.37	0.40	0.32	0.38	0.36
Germacrene D	1487	2.75	2.73	3.07	2.77	2.86	2.83	3.10	2.89	2.95	3.03
Viridiflorene	1497	0.91	0.83	0.91	0.83	0.94	0.97	0.99	0.95	0.94	0.97
Bicyclogermacrene	1503	2.25	2.05	2.26	2.05	2.18	2.15	2.23	2.15	2.17	2.15
Farnesene <(E,E)-*alpha*->	1506	1.36	1.25	1.37	1.25	1.35	1.34	1.4	1.32	1.32	1.35
Bisabolene <*beta*->	1507	0.23	0.21	0.23	0.21	0.23	0.25	0.25	0.25	0.24	0.26
Cadinene <delta->	1523	0.57	0.81	0.94	0.84	0.92	0.94	1.00	0.97	0.94	0.98
Amorphene <delta->	1512	0.69	0.63	0.68	0.61	0.69	0.71	0.71	0.69	0.68	0.70
Spathulenol	1579	1.32	1.24	1.40	1.20	1.27	1.28	1.34	1.28	1.23	1.26
Globulol	1591	0.43	0.33	0.41	0.38	0.40	0.45	0.45	0.46	0.41	0.45
Cubenol <1.10-di-epi->	1619	0.29	0.29	0.32	0.27	0.27	0.29	0.29	0.31	0.27	0.29
Cadinol <-*alpha*->	1653	1.81	1.80	1.96	1.69	1.72	1.72	1.77	1.75	1.70	1.71
Cadinol <*alpha*->	1653	0.61	0.56	0.65	0.56	0.44	0.51	0.51	0.51	0.49	0.51
Bisabolol oxide B <*alpha*->	1657	7.97^a^	7.57^a^	7.59^a^	7.73^a^	7.26^a^	6.49^a^	6.75^a^	6.50^a^	6.47^a^	6.44^a^
Bisabolone oxide A <*alpha*->	1685	10.44	10.14^a^	11.28^a^	9.67^a^	9.67^a^	9.40^a^	9.45^a^	9.56^a^	9.21^a^	9.41^a^
Chamazulene	1731	2.62	2.77	3.07	2.64	2.96	2.72	2.80	2.68	2.61	2.69
Bisabolol oxide A <*alpha*->	1750	30.97	31.97^a^	30.31^a^	33.16^a^	32.3^a^	31.61^a^	31.44^a^	32.17^a^	32.03^a^	32.06^a^

^*∗*^Designation according to Table 1. IR: retention indices (from temperature programming, using the definition of Van den Dool and Kratz [17]). Values designated with the same superscript letters for the dominant compound do not significantly differ at 5% error (Duncan's test).

**Table 6:** Changes in the chemical composition in solutions of hyssop oil.

Compound	IR	Chemical composition of essential oil (mg/ml)									
		Alcohol solutions					Hexane solutions				
		A^*∗*^	B	C	D	K	A	B	C	D	K
Thujene <*alpha*->	931	0.39	0.38	0.39	0.41	0.41	0.41	0.43	0.42	0.44	0.43
Pinene <*alpha*->	939	1.26	1.25	1.26	1.34	1.33	1.33	1.41	1.38	1.43	1.39
Sabinene	975	2.30	2.26	2.26	2.39	2.33	2.32	2.43	2.41	2.48	2.40
Pinene <*beta*->	939	13.78^a^	13.96^a^	13.73^a^	13.32^a^	13.93^a^	13.62^a^	13.15^a^	13.29^a^	12.51^a^	13.00^a^
Myrcene	994	2.39	2.35	2.40	2.55	2.46	2.44	2.52	2.52	2.58	2.55
Limonene	1030	1.77	1.71	1.72	1.77	1.72	1.75	1.79	1.78	1.75	1.73
Cineole <1,8->	1033	6.23^a^	6.13^a^	5.89^a^	5.75^a^	5.60^a^	5.62^a^	5.76^a^	5.74^a^	5.78^a^	5.55^a^
Ocimene <(Z)-*beta*->	1037	1.01	1.00	1.02	1.08	1.05	1.05	1.09	1.07	1.11	1.10
Terpinene <gamma->	1060	0.20	0.20	0.21	0.22	0.21	0.21	0.22	0.22	0.22	0.23
Linalool	1099	1.42	1.37	1.48	1.57	1.50	1.53	1.64	1.55	1.69	1.72
Thujone <*cis*->	1105	0.20	0.20	0.22	0.12	0.22	0.23	0.26	0.23	0.26	0.27
Thujone <*trans*->	1116	0.18	0.17	0.18	0.19	0.18	0.19	0.21	0.19	0.21	0.21
Pinocarveol <*trans*->	1138	0.42	0.41	0.43	0.46	0.45	0.47	0.44	0.48	0.46	0.46
Pinocarvone	1163	8.20^a^	8.68^a^	8.30^a^	7.85^a^	7.98^a^	8.00^a^	8.21^a^	8.13^a^	8.26^a^	7.75^a^
Pinocamphone <*cis*->	1174	28.52^a^	29.38^a^	28.98^a^	27.85^a^	28.34^a^	28.49^a^	27.17^a^	28.03^a^	27.92^a^	27.28^a^
Pinocamphone <*trans*-2-hydroxy->	1249	0.18	0.17	0.18	0.19	0.19	0.21	0.21	0.21	0.22	0.24
Bourbonene <*beta*->	1390	1.74	1.67	1.71	1.83	1.78	1.77	1.79	1.78	1.83	1.90
Methyl eugenol	1405	0.31	0.30	0.32	0.35	0.35	0.36	0.37	0.36	0.38	0.39
Gurjunene <*alpha*->	1412	0.62	0.60	0.61	0.68	0.66	0.65	0.67	0.66	0.69	0.72
Caryophyllene <E->	1420	1.37	1.32	1.31	1.34	1.43	1.41	1.44	1.42	1.47	1.53
Humulene <*alpha*->	1455	0.28	0.27	0.28	0.30	0.30	0.29	0.30	0.29	0.31	0.32
Aromadendrene <allo->	1461	1.99	1.93	1.95	2.07	2.02	1.98	2.03	1.99	2.06	2.10
Germacrene D	1487	3.00	2.87	2.91	3.06	2.96	2.95	2.96	2.94	2.88	3.02
Bicyclogermacrene	1503	3.18	3.07	3.04	3.16	3.06	3.06	3.07	3.06	3.09	3.20
Cadinene <gamma->	1515	0.46	0.45	0.46	0.50	0.50	0.49	0.50	0.48	0.26	0.53
Elemol	1550	1.73	1.69	1.69	1.85	1.81	1.87	1.93	1.86	1.77	2.00
Spathulenol	1579	0.88	0.86	0.90	0.98	0.96	0.97	1.00	0.97	0.97	1.06
Caryophyllene oxide	1585	0.34	0.33	0.35	0.38	0.38	0.37	0.39	0.38	0.36	0.42
Viridiflorol	1594	0.28	0.27	0.28	0.33	0.32	0.33	0.34	0.33	0.32	0.37
Cadinol <epi-*alpha*->	1640	0.38	0.37	0.41	0.45	0.45	0.48	0.49	0.48	0.48	0.55
Eudesmol <*alpha*->	1654	0.46	0.45	0.49	0.55	0.55	0.54	0.56	0.53	0.55	0.60

^*∗*^Designation according to Table 1. IR: retention indices (from temperature programming, using the definition of Van den Dool and Kratz [17]). Values designated with the same superscript letters for the dominant compound do not significantly differ at 5% error (Duncan's test).

**Table 7:** Changes in the chemical composition in solutions of lemon balm oil.

Compound	IR	Chemical composition of essential oil (mg/ml)									
		Alcohol solutions					Hexane solutions				
		A^*∗*^	B	C	D	K	A	B	C	D	K
Pinene <*alpha*->	939	0.82	0.73	0.79	0.72	0.90	1.01	1.02	1.03	1.00	1.03
Pinene <*beta*->	982	2.87	2.86	2.85	2.78	3.11	3.22	3.23	3.23	3.18	3.30
Limonene	1030	7.17^a^	7.60^a^	7.55^a^	6.75^a^	7.24^a^	7.33^a^	7.43^a^	7.45^a^	7.51^a^	7.36^a^
Ocimene <(Z)-*beta*->	1037	0.64	0.56	0.58	0.59	0.28	0.32	0.32	0.33	0.32	0.33
Linalool	1099	0.67	0.58	0.55	0.67	0.61	0.63	0.64	0.66	0.64	0.65
Citronellal	1152	16.60^a^	16.74^a^	16.87^a^	16.77^a^	16.38^a^	16.74^a^	16.92^a^	17.44^a^	16.88^a^	16.92^a^
Terpinen-4-ol	1178	0.33	0.29	0.14	0.32	0.33	0.28	0.29	0.29	0.28	0.30
Terpineol <*alpha*->	1190	0.40	0.36	0.32	0.40	0.36	0.33	0.34	0.35	0.34	0.32
Citronellol	1227	1.17	1.17±	1.16	1.31	1.21	1.28	1.03	1.33	1.29	1.33
Nerol	1232	11.92^a^	11.14^a^	11.65^a^	11.7^a^	10.90^a^	11.24^a^	11.34^a^	11.61^a^	11.61^a^	11.57^a^
Neral	1239	7.39^a^	7.53^a^	7.83^a^	7.96^a^	7.42^a^	7.85^a^	7.84^a^	7.27^a^	7.51^a^	8.16^a^
Geraniol	1254	5.03^a^	4.47^a^	4.29^a^	4.50^a^	4.88^a^	4.85^a^	5.03^a^	4.75^a^	4.96^a^	4.88^a^
Geranial	1269	7.08^a^	6.78^a^	6.64^a^	7.13^a^	7.12^a^	6.44^a^	6.82^a^	7.02^a^	6.59^a^	6.83^a^
Neryl acetate	1363	0.54	0.58	0.61	0.55	0.52	0.51	0.51	0.54	0.53	0.53
Copaene <*alpha*->	1377	2.26	2.82	2.57	2.80	2.65	2.22	2.27	2.33	2.33	2.28
Caryophyllene <E->	1420	8.40^a^	8.54^a^	8.09^a^	8.10^a^	8.47^a^	8.65^a^	8.77^a^	9.07^a^	8.99^a^	8.48^a^
Bergamotene <alpha-*trans*>	1436	1.71	1.8	1.46	2.01	1.48	1.25	1.28	1.33	1.31	1.29
Humulene <*alpha*->	1455	2.08	2.04	1.82	1.93	1.85	1.51	1.54	1.60	1.58	1.54
Germacrene D	1487	1.14	1.18	1.18	1.19	1.18	0.91	0.91	0.93	0.95	0.90
Cadinene <gamma->	1515	0.56	0.58	0.46	0.60	0.45	0.34	0.35	0.37	0.38	0.35
Cadinene <delta->	1525	1.17	1.11	0.97	1.18	1.04	0.85	0.86	0.90	0.89	0.86
Caryophyllene oxide	1585	0.92	0.99	0.77	1.15	0.78	0.58	0.60	0.59	0.59	0.56

^*∗*^Designation according to Table 1. IR: retention indices (from temperature programming, using the definition of Van den Dool and Kratz [17]). Values designated with the same superscript letters for the dominant compound do not significantly differ at 5% error (Duncan's test).

**Table 8:** Changes in the chemical composition in solutions of rosemary oil.

Compound	IR	Chemical composition of essential oil (mg/ml)									
		Alcohol solutions					Hexane solutions				
		A^*∗*^	B	C	D	K	A	B	C	D	K
Pinene <*alpha*->	939	6.31^a^	6.30^a^	6.45^a^	6.27^a^	6.32^a^	7.13^a^	7.30^a^	7.28^a^	7.32^a^	6.98^a^
Camphene	953	5.22^a^	5.22^a^	5.33^a^	5.21^a^	5.26^a^	5.69^a^	5.83^a^	5.77^a^	5.74^a^	5.75^a^
Pinene <*beta-*>	981	3.76	3.74	3.77	3.73	3.72	4.29	4.34	4.37	4.31	4.27
Myrcene	994	1.86	1.87	1.91	1.89	1.89	2.5	2.56	2.53	2.45	2.41
Phellandrene <*alpha*->	1008	0.64	0.63	0.64	0.64	0.63	0.63	0.65	0.65	0.64	0.63
Terpinene <*alpha*->	1020	0.55	0.54	0.57	0.55	0.55	0.42	0.44	0.43	0.43	0.42
Cymene <ortho->	1028	5.69^a^	5.28^a^	5.35^a^	4.46^a^	6.01^a^	7.09^a^	6.99^a^	6.53^a^	6.32^a^	5.59^a^
Cineole <1,8->	1033	20.31^a^	20.13^a^	20.75^a^	20.39^a^	20.38^a^	19.54^a^	19.16^a^	19.06^a^	19.22^a^	19.47^a^
Terpinene <gamma->	1060	2.17	2.17	2.18	2.18	2.05	2.12	2.15	2.15	2.11	2.21
Linalool	1099	6.24^a^	6.23^a^	6.29^a^	6.37^a^	6.06^a^	6.02^a^	5.95^a^	6.08^a^	6.03^a^	6.22^a^
Pinene oxide <*alpha*->	1103	0.36	0.35	0.36	0.36	0.35	0.36	0.37	0.37	0.36	0.38
Camphor	1146	13.80^a^	13.75^a^	13.92^a^	13.93^a^	13.61^a^	12.94^a^	12.70^a^	13.02^a^	12.85^a^	13.36^a^
Isoborneol	1159	2.53	2.53	2.52	2.57	2.49	2.25	2.26	2.24	2.22	2.33
Borneol	1169	3.73	3.69	3.74	3.78	3.70	3.83	3.55	3.77	3.62	3.91
Terpineol <*alpha*->	1190	2.87	2.79	2.83	2.90	2.83	2.84	2.87	2.86	2.86	2.99
Terpineol <gamma->	1203	1.47	1.46	1.47	1.50	1.42	1.25	1.27	1.23	1.23	1.29
Carvone	1243	0.82	0.81	0.82	0.85	0.79	0.76	0.78	0.77	0.77	0.79
Isobornyl acetate	1287	2.50	2.44	2.49	2.53	2.43	2.37	2.39	2.35	2.36	2.44
Caryophyllene <E->	1420	8.38^a^	8.26^a^	7.84^a^	9.06^a^	7.86^a^	7.06^a^	7.25^a^	7.12^a^	7.19^a^	7.51^a^

^*∗*^Designation according to Table 1. IR: retention indices (from temperature programming, using the definition of Van den Dool and Kratz [17]). Values designated with the same superscript letters for the dominant compound do not significantly differ at 5% error (Duncan's test).

**Table 9:** Changes in chemical composition of standard solutions.

Compound	IR	Concentration (mg/ml)									
		Alcohol solutions					Hexane solutions				
		A^*∗*^	B	C	D	K	A	B	C	D	K
Limonene	1030	1.46^a^	1.45^a^	1.51^a^	1.48^a^	1.49^a^	1.48^a^	1.47^a^	1.50^a^	1.51^a^	1.46^a^
Cineole <1,8->	1033	1.47^a^	1.51^a^	1.47^a^	1.49^a^	1.51^a^	1.52^a^	1.53^a^	1.54^a^	1.47^a^	1.49^a^
Terpinene <gamma->	1060	1.46^a^	1.46^a^	1.51^a^	1.55^a^	1.52^a^	1.47^a^	1.45^a^	1.48^a^	1.53^a^	1.54^a^
Linalool	1099	1.45^a^	1.45^a^	1.48^a^	1.51^a^	1.49^a^	1.50^a^	1.48^a^	1.47^a^	1.49^a^	1.50^a^
Menthone	1155	1.50^a^	1.47^a^	1.46^a^	1.48^a^	1.45^a^	1.46^a^	1.50^a^	1.47^a^	1.53^a^	1.47^a^

^*∗*^Designation according to Table 1. Values designated with the same superscript letters for the dominant compound do not significantly differ at 5% error (Duncan's test). IR: retention indices (from temperature programming, using the definition of Van den Dool and Kratz [17]).

## Data Availability

The data used to support the findings of this study are included within the article.
